# The importance of the bow and lean test as an initial positional test for diagnosing BPPV

**DOI:** 10.1007/s00405-025-09512-8

**Published:** 2025-06-14

**Authors:** Ercan Karababa, Sanem Can Sarıoğlu, Fatma Ceyda Akın Öçal, Deniz Uğur Cengiz, Mehmet Arslan, Bülent Satar

**Affiliations:** 1 Gulhane Faculty of Health Sciences, Department of Audiology, University of Health Sciences, Ankara, Türkiye; 2 Faculty of Health Sciences, Department of Audiology, İnönü University, Malatya, Türkiye; 3 Department of Otorhinolaryngology, University of Health Sciences, Gulhane Training and Research Hospital, Ankara, Türkiye; 4 Faculty of Medicine, Department of Otorhinolaryngology, İnönü University, Malatya, Türkiye; 5 University of Health Sciences, Gulhane Faculty of Medicine, Department of Otorhinolaryngology, Ankara, Türkiye

**Keywords:** Bow and lean test, Dix hallpike test, Head roll test, Posterior canal, Positional vertigo

## Abstract

**Purpose:**

This study aimed to evaluate the diagnostic potential of nystagmus direction observed during bow and lean tests (BLTs) in patients with posterior canal canalolithiasis BPPV (P-BPPV), lateral canal canalolithiasis BPPV (Lca-BPPV), and lateral canal cupulolithiasis BPPV (Lcu-BPPV).

**Methods:**

A total of 62 patients (40 women, 22 men; aged 24–70 years) with clinically suspected BPPV were enrolled. Diagnoses included 39 cases of P-BPPV, 15 of Lca-BPPV, and 8 of Lcu-BPPV. Each participant underwent the Dix-Hallpike Test (DH), Head Roll Test (HRT), and bow and lean tests for diagnostic assessment. The primary outcome was the presence and direction of nystagmus during BLTs, in relation to BPPV subtype.

**Results:**

Nystagmus was detected in 77.4% of subjects during the bow test and in 46.8% during the lean test. A statistically significant difference was found in nystagmus direction across BPPV subtypes (*p* < 0.05). Right-beating horizontal nystagmus during the bow test was significantly more frequent in right-sided Lca-BPPV. Right down-beating torsional nystagmus during the bow test occurred exclusively in left P-BPPV, while left down-beating torsional nystagmus was seen only in right P-BPPV. Right up-beating torsional nystagmus was significantly associated with right P-BPPV, and left up-beating torsional nystagmus with left P-BPPV (*p* < 0.05).

**Conclusions:**

In P-BPPV, DH-induced nystagmus direction was opposite in the bow position but matched in the lean position. These findings underscore the diagnostic value of BLTs, particularly the bow test, in identifying the affected canal in posterior and lateral canal BPPV.

## Introduction

Benign paroxysmal positional vertigo (BPPV) is among the most prevalent etiologies of peripheral vertigo, distinguished by brief episodes of rotational vertigo precipitated by head position [1]. This phenomenon arises from the displacement of otoconia that have become detached from the utricular otolithic membrane, subsequently migrating towards the semicircular canals (SCCs). The severity, frequency, and duration of the clinical manifestations of BPPV are subject to variation depending on the characteristics of the affected SCC and the location of the autolytic particles. The diagnosis of BPPV is typically made on the basis of characteristic clinical findings, including latency, reversibility, crescendo effect, transience of symptoms, and fatigue. A detailed clinical history is usually obtained, and specific diagnostic maneuvers are applied, in order to arrive at an accurate diagnosis [2].

The Bow and Lean Tests (BLTs), originally developed by Cohoung et al. in 2006, was designed to identify the affected canal in patients diagnosed with BPPV of the lateral canal type. The test procedure involves having the patient tilt their head 90° forward while in a seated position, which is referred to as the “bow test.” The direction of nystagmus observed during this test aligns with the direction of the affected canal in cases of lateral canal canalolithiasis (Lca-BPPV). The test is referred to as the “lean test” when the head is tilted back 45°. The direction of nystagmus observed in the lean test is consistent with the affected canal in cases of lateral canal cupulolithiasis (Lcu-BPPV) [3]. The utilization of BLTs has been demonstrated to be a reliable method for identifying the labeled channel in cases of Lca-BPPV and Lcu-BPPV [4]. There is a paucity of studies on the nystagmus characteristics of BLTs applied to patients diagnosed with P-BPPV [5–7]. In a study in which BLTs was applied in posterior and lateral canal BPPV, in patients with posterior canal canalolithiasis (P-BPPV), the direction of nystagmus triggered by the Dix-Hallpike test (DH) on the affected side was observed to be the same in the lean position and opposite (reverse nystagmus) in the bow position [5]. Additionally, it has been documented that the presence of vertical nystagmus in patients undergoing BLTs may be indicative of P-BPPV involvement [7]. While the BLTs position is considered appropriate for the lateral SSC plane, it has the potential to stimulate the posterior canal plane as well [5].

Although BPPV is classified as benign, it is regarded as a clinically significant disorder due to the recurrent nature of the symptoms and the adverse impact on quality of life [8]. Consequently, an accurate diagnosis of BPPV is imperative, as it dictates the duration of treatment. Although the effectiveness of the BLTs in identifying the affected side in cases of lateral BPPV has been well documented, its role in the diagnosis of P-BPPV remains to be fully elucidated. Therefore, the objective of this study was to investigate the diagnostic role of nystagmus directions observed during BLTs in posterior and lateral canal BPPV.

## Materials and methods

The present study was approved by the ethics committee of the university (Decision No. 2024/6659), and it was conducted in accordance with the Declaration of Helsinki. The present study was executed in accordance with established ethical principles, and all participants provided written and detailed informed consent. All subjects underwent neuro-otological evaluation. The present study included individuals who met the diagnostic criteria for P-BPPV, Lca-BPPV, and Lcu-BPPV, as established by the Barany Society [9]. The study population comprised 62 individuals between the ages of 24 and 70 years, including 39 individuals diagnosed with P-BPPV, 15 individuals diagnosed with Lca-BPPV, and 8 individuals diagnosed with Lcu-BPPV. The ICS Chart 200 (GN Otometrics, Taastrup, Denmark), also known as the videonystagmography (VNG) test, was utilized to assess the vestibular function of the patients. Subjects diagnosed with vertigo were fitted with VNG goggles, and their eye movements were recorded using infrared camera goggles. Spontaneous nystagmus, gaze tests, positional tests, and BLTs were performed in a dark room. Patients diagnosed with DH or head roll test (HRT) underwent BLTs with video-glasses before reposition maneuvering. The direction of nystagmus observed in patients with BPPV was documented. The inclusion criteria encompassed individuals diagnosed with P-BPPV, Lca-BPPV, or Lcu-BPPV. Furthermore, subjects exhibiting no nystagmus and an amelioration of dizziness symptoms during DH and HRT were included in the study, as determined during the control examination conducted one week after the repositioning maneuver. This study employed an approach to evaluate the direction of nystagmus during BLTs, without comparing the severity of nystagmus observed in DH, HRT, and BLTs.

Exclusion criteria encompassed multicanal BPPV, a history of ear surgery, restriction of head and neck movements, sudden sensorineural hearing loss, Meniere’s disease, and neurological diseases.

### Statistical analysis

The data utilized in the study were subjected to analysis using the SPSS 28.0 statistical software program. Descriptive statistics for continuous variables were calculated as mean, standard deviation, minimum, and maximum values. The descriptive statistics of the categorical variables were summarized using frequency (n) and percentage (%) values. In the analysis process, cross-tabulations were created to examine the relationships between variables, and a chi-square test was applied. Additionally, histogram graphs were used to visually present the distribution of the data.

## Results

A total of 62 subjects were included in the study; 40 (64.5%) of them were female and 22 (35.5%) were male. The age range of the participants was between 24 and 70 years, with a mean age of 48.84 ± 11.42 years.

The distribution of the individuals included in the study according to the affected SCC is as follows: right P-BPPV cases 24 (38.7%), left P-BPPV cases 15 (24.2%), right Lca-BPPV cases 9 (14.5%), right Lcu-BPPV cases 5 (8.1%), left Lca-BPPV cases 6 (9.7%) and left Lcu-BPPV cases 3 (4.8%) (Table 1).

**Table 1:** Distribution of affected semicircular canal involvement

Variable	Groups	Frequency	Percent
Affected Side (SCC)	Right Posterior Canal Canalolithiasis	24	38.7
	Left Posterior Canal Canalolithiasis	15	24.2
	Right Lateral Canal Canalolithiasis	9	14.5
	Right Lateral Canal Cupulolithiasis	5	8.1
	Left Lateral Canal Canalolithiasis	6	9.7
	Left Lateral Canal Cupulolithiasis	3	4.8
Total		62	100

In this study, nystagmus assessment during BLTs revealed important findings. Among all subjects, 14 participants (22.6%) had no nystagmus during the bow test, while 48 participants (77.4%) had nystagmus. During the lean test, 33 participants (53.2%) had no nystagmus, while 29 participants (46.8%) had nystagmus. Moreover, when both tests were evaluated collectively, 39 subjects (62.9%) exhibited no nystagmus in either the bow or lean tests, while 23 subjects (37.1%) demonstrated nystagmus in both tests (Table 2).

**Table 2:** Frequency and distribution of nystagmus in the bow and lean tests

Variable	Nystagmus	Frequency	Percent
Bow Test	No	14	22.6
	Yes	48	77.4
Lean Test	No	33	53.2
	Yes	29	46.8
Bow and Lean Test	No	39	62.9
	Yes	23	37.1
Total		62	100

**Fig. 1:**
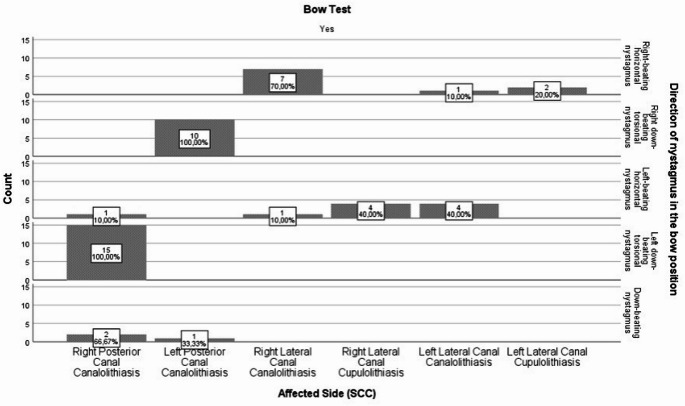
Affected semicircular canal and directional distribution of nystagmus in the bow test

**Fig. 2:**
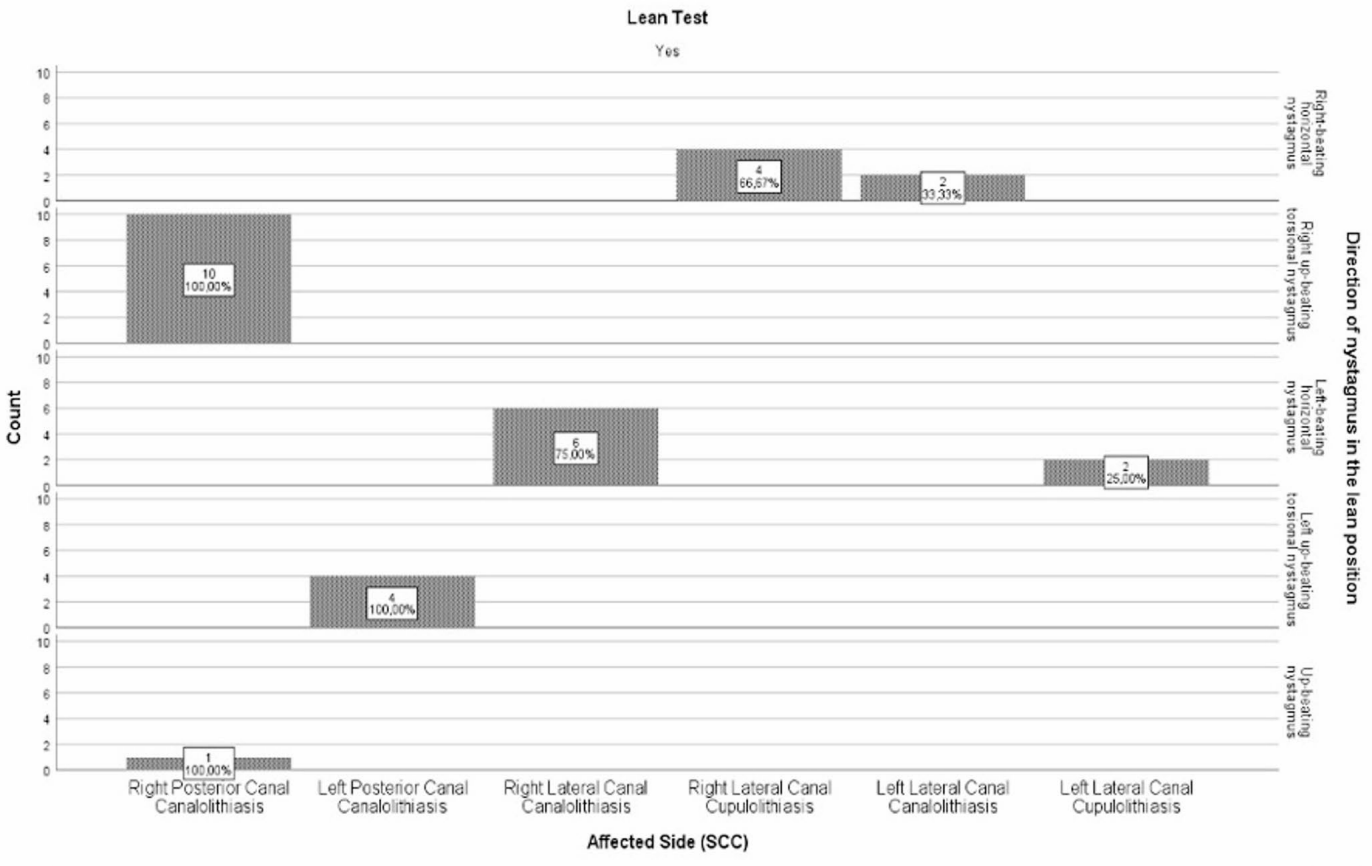
Affected semicircular canal and directional distribution of nystagmus in the lean test.

In patients diagnosed with posterior and lateral canal BPPV, the nystagmus directions observed during the bow test are depicted in Fig. 1, while the nystagmus directions observed during the lean test are illustrated in Fig. 2.

A statistically significant relationship was identified between the affected SCCs in terms of the frequency of nystagmus directions observed during BLTs (*p* < 0.05). (Table 3 and Table 4).

**Table 3:** Distribution of affected semicircular canals and nystagmus direction according to the bow test

Nystagmus		Affected SCC						Total	χ^2^	Sig. (p)
		Right P-BPPV	Left P-BPPV	Right Lca-BPPV	Right Lcu-BPPV	Left Lca-BPPV	Left Lcu-BPPV			
Right-beating horizontal nystagmus	**n**	0	0	7	0	1	2	**10**	10.972	0.001*
	**%**	0	0	70	0	10	20	**100%**		
Right down-beating torsional nystagmus	**n**	0	10	0	0	0	0	**10**		
	**%**	0	100	0	0	0	0	**100%**		
Left-beating horizontal nystagmus	**n**	1	0	1	4	4	0	**10**		
	**%**	10	0	10	40	40	0	**100%**		
Left down-beating torsional nystagmus	**n**	15	0	0	0	0	0	**15**		
	**%**	100	0	0	0	0	0	**100%**		
Down-beating nystagmus	**n**	2	1	0	0	0	0	**3**		
	**%**	66.7	33.3	0	0	0	0	**100%**		
Total	**n**	**18**	**11**	**8**	**4**	**5**	**2**	**48**		
	**%**	**37.5**	**22.9**	**16.7**	**8.3**	**10.4**	**4.2**	**100%**		

n; frequency, %; percent, χ^2^; Chi Square Test, SCC; Semicircular canal, *p < 0,05; There is a statistical difference between groups, Right P-BPPV; Right Posterior Canal Canalolithiasis, Left P-BPPV; Left Posterior Canal Canalolithiasis, Right Lca-BPPV; Right Lateral Canal Canalolithiasis, Right Lcu-BPPV; Right Lateral Canal Cupulolithiasis, Left Lca-BPPV; Left Lateral Canal Canalolithiasis, Left Lcu-BPPV; Left Lateral Canal Cupulolithiasis

**Table 4:** Distribution of affected semicircular canals and nystagmus direction according to the lean test

Nystagmus		Affected SCC						Total	χ^2^	Sig. (p)
		Right P-BPPV	Left P-BPPV	Right Lca-BPPV	Right Lcu-BPPV	Left Lca-BPPV	Left Lcu-BPPV			
Right-beating horizontal nystagmus	**n**	0	0	0	4	2	0	**6**	76.687	0.001*
	**%**	0	0	0	66.7	33.3	0	**100%**		
Right up-beating torsional nystagmus	**n**	10	0	0	0	0	0	**10**		
	**%**	100	0	0	0	0	0	**100%**		
Left-beating horizontal nystagmus	**n**	0	0	6	0	0	2	**8**		
	**%**	0	0	75	0	0	25	**100%**		
Left up-beating torsional nystagmus	**n**	0	4	0	0	0	0	**4**		
	**%**	0	100	0	0	0	0	**100%**		
Up-beating nystagmus	**n**	1	0	0	0	0	0	**1**		
	**%**	100	0	0	0	0	0	**100%**		
Total	**n**	**11**	**4**	**6**	**4**	**2**	**2**	**29**		
	**%**	**37.9**	**13.8**	**20.7**	**13.8**	**6.9**	**6.9**	**100%**		

n; frequency, %; percent, **χ**^**2**^; Chi Square Test, SCC; Semicircular canal, *p < 0,05; There is a statistical difference between groups, Right P-BPPV; Right Posterior Canal Canalolithiasis, Left P-BPPV; Left Posterior Canal Canalolithiasis, Right Lca-BPPV; Right Lateral Canal Canalolithiasis, Right Lcu-BPPV; Right Lateral Canal Cupulolithiasis, Left Lca-BPPV; Left Lateral Canal Canalolithiasis, Left Lcu-BPPV; Left Lateral Canal Cupulolithiasis

In the bow test, which was performed on a total of 62 individuals, 10 instances (100%) of right-beating horizontal nystagmus were identified. Of these 10 cases, 7 (70%) were diagnosed as right Lca-BPPV, 1 (10%) as left Lca-BPPV, and 2 (20%) as left Lcu-BPPV. A statistically significant difference in the prevalence of right-beating horizontal nystagmus was observed between Right Lca-BPPV cases and left Lca-BPPV and left Lcu-BPPV cases (*p* < 0.05). During the bow test, 10 (100%) cases with right down-beating torsional nystagmus were identified. All of these cases (100%) were diagnosed as left P-BPPV. Nystagmus was found to be statistically significantly higher in the left P-BPPV group compared to the other groups (*p* < 0.05) (Table 3).

In the bow test, 10 (100%) cases with left-beating horizontal nystagmus were identified. Of these cases, 1 (10%) was diagnosed as right P-BPPV, 1 (10%) as right Lca-BPPV, 4 (40%) as right Lcu-BPPV, and 4 (40%) as left Lca-BPPV. The frequency of left-beating horizontal nystagmus observed in right Lcu-BPPV cases was found to be statistically significantly higher compared to right P-BPPV and left P-BPPV cases (*p* < 0.05). Conversely, the prevalence of left-beating horizontal nystagmus was found to be considerably higher in cases of left Lca-BPPV compared to right P-BPPV and left P-BPPV cases (*p* < 0.05). Given that the frequency of left-beating horizontal nystagmus observed in the right Lcu-BPPV and left Lca-BPPV groups was equivalent, it was determined that there was no statistical difference between the two groups (*p* > 0.05) (Table 3).

In the bow test, 15 cases (100%) of left down-beating torsional nystagmus were identified. All of these cases were diagnosed with right P-BPPV. The prevalence of nystagmus in the right P-BPPV group was found to be statistically significantly higher compared to the other groups (*p* < 0.05) (Table 3).

The results of the examination indicated that 3 (100%) of the cases presented with down-beating nystagmus. Of these cases, 2 (66.7%) were identified as right P-BPPV, and 1 (33.3%) as left P-BPPV. The occurrence of down-beating nystagmus was found to be statistically higher between cases of right P-BPPV and left P-BPPV (*p* < 0.05) (Table 3).

In the lean test, 6 (100%) cases with right-beating horizontal nystagmus were identified. Of these cases, 4 (66.7%) were diagnosed as right Lcu-BPPV and 2 (33.3%) as left Lca-BPPV. The frequency of right-beating horizontal nystagmus observed in right Lcu-BPPV cases was statistically higher compared to left Lca-BPPV cases (*p* < 0.05) (Table 4).

Lean testing identified 10 (100%) cases with right up-beating torsional nystagmus. All of these cases (100%) were diagnosed with right P-BPPV. The observation of nystagmus only in the right P-BPPV group was statistically significantly higher compared to the other groups (*p* < 0.05) (Table 4).

In the lean test, 8 (100%) cases with left-beating horizontal nystagmus were identified. Of these cases, 6 (75%) were diagnosed as right Lca-BPPV and 2 (25%) as left Lcu-BPPV. The frequency of left-beating horizontal nystagmus observed in right Lca-BPPV cases was found to be statistically significantly higher compared to left Lcu-BPPV cases (*p* < 0.05) (Table 4).

On the lean test, 4 cases (100%) with left up-beating torsional nystagmus were identified. All of these cases (100%) were diagnosed as left P-BPPV. The observation of nystagmus only in the left P-BPPV group was statistically significantly higher compared to the other groups (*p* < 0.05) (Table 4).

Lean test identified 1 case (100%) with up-beating nystagmus. This case (100%) was diagnosed as right P-BPPV. The observation of nystagmus only in the right P-BPPV group was statistically significantly higher compared to the other groups (*p* < 0.05) (Table 4).

The direction of nystagmus seen in the affected canal in P-BPPV patients was opposite to the nystagmus observed in the bow test and the same as in the lean test. Assuming this as a criterion to confirm the diagnosis of P-BPPV, according to Tables 1 and 3, the diagnostic value of the bow test in identifying the affected canal in patients with P-BPPV is 64.1% (25 of 39). According to Tables 1 and 4, the diagnostic value of the lean test in identifying the affected canal is 35.89% (14 of 39).

The diagnosis is considered confirmed when the nystagmus direction observed in the affected Lca-BPPV is in the same direction as the bow test and the nystagmus direction observed in the affected Lcu-BPPV is in the same direction as the lean test. Taking this into account in our study, according to Tables 1 and 3, the diagnostic accuracy of the bow test in Lca-BPPV was 73.33% (11 of 15) and according to Tables 1 and 4, the diagnostic accuracy of the lean test in Lcu-BPPV was 75% (6 of 8).

## Discussion

In this study, we evaluated the role of nystagmus directions observed during BLTs in identifying the affected canal in the diagnosis of P-BPPV, Lca-BPPV, and Lcu-BPPV cases. The findings of the present study indicated that the direction of nystagmus in the bow test, as opposed to the lean test, may serve as a significant indicator in determining the affected canal in P-BPPV. Furthermore, BLTs demonstrated a higher diagnostic accuracy in patients with Lca-BPPV and Lcu-BPPV compared to P-BPPV.

Choi et al. emphasized the role of BLTs in the diagnosis of P-BPPV and Lca-BPPV and reported that leaning nystagmus was in the same direction and bowing nystagmus was in the opposite direction especially in P-BPPV in accordance with the DH test [5]. In our study, the direction of nystagmus observed in the bow test in individuals diagnosed with P-BPPV was opposite to that in the DH test, whereas the direction in the lean test was the same. However, it should be noted that, in contrast to the study by Choi et al., our study evaluated the diagnostic accuracy of BLTs with numerical data. The findings of the present study indicate that the bow test exhibited a diagnostic accuracy of 64.1%, while the lean test demonstrated a diagnostic accuracy of 35.89% in the identification of the affected channel in P-BPPV. Furthermore, Choi et al. reported that the diagnostic yield of BLTs in identifying the affected side in patients with Lca-BPPV was 74%. However, they observed that BLTs was not sufficient to distinguish between Lcu-BPPV and light cupula cases due to persistent nystagmus. In our study, it was observed that bow test had an accuracy rate of 73.33% for Lca-BPPV and lean test had an accuracy rate of 75% for Lcu-BPPV. These findings reveal the importance of bow test especially in the diagnosis of P-BPPV, while BLTs may have a varying diagnostic value in lateral canal BPPV (Cupulolithiasis and Light Cupula).

The usefulness of the BTL test as a diagnostic tool for the accurate identification of the affected SCC in BPPV is an area of active research, with conflicting results across studies. As demonstrated by Martin-Sanz et al., the utilization of BLTs is not limited to the diagnosis of lateral canal BPPV; its application extends to other variants of P-BPPV. In their study, the researchers reported that the vertical nystagmus observed during BLTs may be a significant marker for P-BPPV. They further noted that the direction of nystagmus differs between P-BPPV subtypes, including posterior cupulolithiasis, short-arm canalolithiasis, and non-ampullary arm canalolithiasis [7]. The study by Martinz-Sanz et al. is distinct from our own in that it incorporates a range of P-BPPV types. However, it shares similarities with our study in that a downward nystagmus was observed in patients with P-BPPV. In their study, the researchers observed down beat nystagmus in approximately 86% of patients with P-BPPV (canalolithiasis group) in the bow test, while up beating nystagmus was observed in approximately 30% of patients in the lean test. This finding is higher than the torsional down beating nystagmus seen in the bow test in our study (64.1%) and similar to the torsional up beating nystagmus seen in the lean test (35, 89%). One hypothesis for this discrepancy is that the difference in the nystagmus seen in the bow test from our study may be due to the different severity of nystagmus seen in the patients and the time of BLT application.

Choo et al. also drew attention to the presence of P-BPPV in patients with vertical nystagmus during BLTs and suggested that this nystagmus may reveal “hidden” P-BPPV. It was reported that vertigo complaints persisted, particularly among individuals with vertical nystagmus on BLTs. However, no nystagmus was observed on DH in the follow-up examination after repositioning the subject. It was suggested that the vertical nystagmus observed during BLTs may occur in patients with “hidden P-BPPV” who complain of remnant vertigo symptoms. In summary, the research indicated that the vertical nystagmus observed in BLTs may be indicative of the persistence of otoconial debris within the posterior SCC [6]. Although Choo et al. observed vertical nystagmus in patients with P-BPPV, they also observed vertical nystagmus in the lateral BPPV group. In contrast, our study did not observe any cases of vertical nystagmus in patients with Lca-BPPV and Lcu-BPPV. Choo et al. suggested that the vertical nystagmus seen in lateral canal BPPV may be seen in patients with both posterior and lateral canal involvement.

To the best of our knowledge, none of the studies reported in the literature provide information on the torsional characteristics of the nystagmus observed in the bow test in P-BPPV. The nystagmus seen in BLTs is usually described as down-beating or up-beating. In this study, the reverse pattern of nystagmus observed in the DH was replicated in the bow test, particularly in the affected posterior SCC. To illustrate, a patient with right up-beating torsional nystagmus in the DH exhibited left down-beating torsional nystagmus in the bow test. In the case of classic P-BPPV, when the patient is in the sitting position, the otoconia are typically located in the anterior part of the long arm. In the case of P-BPPV, when the patient is positioned in a bow-like stance, a potential mechanism involves the movement of the otoconia toward the ampulla, leading to ampullopetal flow. This will result in inhibition in posterior SCC, leading to a downward torsional direction of nystagmus [6]. In the lean position, the otoconia may remain immobilized or move away from the ampulla to form ampullofugal flow because the backward movement of the head is limited. If the otoconia remain stationary, no nystagmus is observed. However, if they move away from the ampulla, upward torsional nystagmus compatible with the affected canal may be observed, as there will be excitation in the posterior SCC [5, 6]. In the present study, nystagmus consistent with SCC was not observed in all patients with BPPV types in the BLTs due to the density and position of the otoconia in the plane of the canal.

Kale et al. evaluated the efficacy of BLTs in lateral canal BPPV and examined the confirmation rates of the tests for the affected SCC, especially in differentiating canalolithiasis and cupulolithiasis. In their study, the researchers found that the BTLs exhibited a high diagnostic accuracy rate for lateral canal cupulolithiasis (87.5%), though this rate was lower for cases of canalolithiasis (60%) [10]. In a similar vein, Lee et al. reported a higher diagnostic accuracy of the BLTs in patients with Lcu-BPPV [11]. The findings of both studies demonstrate congruence with the present results in terms of higher diagnostic accuracy rates in patients with Lcu-BPPV. However, the disparities in diagnostic accuracy rates are pronounced. The potential causes of these variations may encompass the following factors: variations in the rate at which clinicians administer BLTs and HRT, differences in head flexion angle, and individual variations in nystagmus severity.

Overall, our study reveals that BLTs can be a guide not only in the diagnosis of lateral canal BPPV but also in the diagnosis of posterior canal BPPV and emphasizes the diagnostic value of the bow test especially in patients with P-BPPV. In light of the comparatively diminutive nature of our sample size in contrast to that of the studies conducted by Martinz-Sanz et al. and Choo et al., the necessity of corroboration through larger-scale investigations becomes imperative.

Our general recommendation is that in patients presenting with positional vertigo symptoms, performing the bow and lean test prior to the DH and HRT tests may provide preliminary guidance on the appropriate choice of diagnostic positional test, based on the direction and torsional characteristics of the observed nystagmus.

## Conclusions

Our current study demonstrated the diagnostic importance of the nystagmus directions observed during BLTs in individuals diagnosed with P-BPPV, Lca-BPPV and Lcu-BPPV. In particular, the direction of nystagmus observed during BLTs indicates that it is more effective in terms of diagnostic accuracy for Lca-BPPV and Lcu-BPPV types. However, it may also serve as a determining test in the diagnosis of P-BPPV.
